# Author Correction: An Open Source Python Library for Anonymizing Sensitive Data

**DOI:** 10.1038/s41597-024-04283-z

**Published:** 2024-12-20

**Authors:** Judith Sáinz-Pardo Díaz, Álvaro López García

**Affiliations:** Instituto de Física de Cantabria (IFCA), CSIC-UC Avda. los Castros s/n, 39005 Santander, Spain

**Keywords:** Computer science, Software, Scientific data

Correction to: *Scientific Data* 10.1038/s41597-024-04019-z, published online 26 November 2024

In this article the acknowledgements section was incorrect. It currently reads ‘The authors would like to thank the funding through the project AI4EOSC “Artificial Intelligence for the European Open Science Cloud” that has received funding from the European Union’s Horizon Europe research and innovation programme under grant agreement number 101058593 and from the SIESTA project “Secure Interactive Environments for Sensitive daTa Analytics”, funded by the European Union (Horizon Europe) under grant agreement number 101131957’ and should read, ‘The authors would like to thank the funding through the SIESTA project “Secure Interactive Environments for Sensitive daTa Analytics”, funded by the European Union (Horizon Europe) under grant agreement number 101131957’. The original article has been corrected.

